# Integrating an internet-mediated walking program into family medicine clinical practice: a pilot feasibility study

**DOI:** 10.1186/1472-6947-11-47

**Published:** 2011-06-24

**Authors:** David E Goodrich, Lorraine R Buis, Adrienne W Janney, Megan D Ditty, Christine W Krause, Kai Zheng, Ananda Sen, Victor J Strecher, Michael L Hess, John D Piette, Caroline R Richardson

**Affiliations:** 1 Department of Family Medicine, University of Michigan, 1018 Fuller St, Ann Arbor, 48104, USA; 2 VA Center for Clinical Management Research, VA Ann Arbor Healthcare System, 2215 Fuller Rd, Mailstop 152, Ann Arbor, MI 48113, USA; 3 Wayne State University, College of Nursing - Adult Health, 5557 Cass Ave Room 368 Cohn Bldg, Detroit, 48202, USA; 4 School of Public Health, Department of Health Management and Policy, University of Michigan, M3531 SPH II, Ann Arbor, 48109, USA; 5 Department of Statistics, University of Michigan, 439 West Hall, 1085, South University Ave, Ann Arbor, 48109, USA; 6 Schools of Medicine and Public Health, University of Michigan, 5D04 300 NIB, Ann Arbor, 48109, USA; 7 HealthMedia, 130 South First St, Ann Arbor, 48104, USA; 8 Department of Internal Medicine, University of Michigan Medical School, 300 N. Ingalls Building, Room 7E10, Ann Arbor, 48109, USA; 9 Michigan Diabetes Research and Training Center, Brehm Tower 6414, 1000 Wall St, Ann Arbor, 48105, USA

**Keywords:** Pedometer, family medicine, Internet-mediated intervention, physical activity, walking, primary care, patient-centered medical home

## Abstract

**Background:**

Regular participation in physical activity can prevent many chronic health conditions. Computerized self-management programs are effective clinical tools to support patient participation in physical activity. This pilot study sought to develop and evaluate an online interface for primary care providers to refer patients to an Internet-mediated walking program called Stepping Up to Health (SUH) and to monitor participant progress in the program.

**Methods:**

In Phase I of the study, we recruited six pairs of physicians and medical assistants from two family practice clinics to assist with the design of a clinical interface. During Phase II, providers used the developed interface to refer patients to a six-week pilot intervention. Provider perspectives were assessed regarding the feasibility of integrating the program into routine care. Assessment tools included quantitative and qualitative data gathered from semi-structured interviews, surveys, and online usage logs.

**Results:**

In Phase I, 13 providers used SUH and participated in two interviews. Providers emphasized the need for alerts flagging patients who were not doing well and the ability to review participant progress. Additionally, providers asked for summary views of data across all enrolled clinic patients as well as advertising materials for intervention recruitment. In response to this input, an interface was developed containing three pages: 1) a recruitment page, 2) a summary page, and 3) a detailed patient page. In Phase II, providers used the interface to refer 139 patients to SUH and 37 (27%) enrolled in the intervention. Providers rarely used the interface to monitor enrolled patients. Barriers to regular use of the intervention included lack of integration with the medical record system, competing priorities, patient disinterest, and physician unease with exercise referrals. Intention-to-treat analyses showed that patients increased walking by an average of 1493 steps/day from pre- to post-intervention (*t *= (36) = 4.13, *p *< 0.01).

**Conclusions:**

Providers successfully referred patients using the SUH provider interface, but were less willing to monitor patient compliance in the program. Patients who completed the program significantly increased their step counts. Future research is needed to test the effectiveness of integrating SUH with clinical information systems over a longer evaluation period.

## Background

Physical activity is recommended as first-line medical therapy for preventing and managing many chronic diseases [1-4]. Moderate-intensity physical activity such as walking can reduce the risk of developing chronic diseases, particularly among individuals at high risk for type 2 diabetes and cardiovascular disease [5-10]. However, there are few effective interventions for promoting physical activity in the primary care setting [11,12].

Medical guidelines emphasize the active role primary care physicians (PCPs) should play in helping patients initiate and sustain physical activity programs. Provider recommendations can help motivate patients to initiate an exercise program. PCPs can also be instrumental helping patients safely re-engage in activity after an illness or medical issue [9,13,14]. In practice, a provider's ability to promote physical activity has been limited by time constraints, lack of training in exercise prescription, concerns over monitoring patient safety, and lack of access to cost-effective resources that help patients remain active [11,12,15-17]. Internet-based, computer-tailored intervention programs may make it easier for providers to support patients with personalized self-management information and encouragement and thus facilitate sustained adherence to physical activity [18-20].

We previously developed and tested Stepping Up to Health (SUH), an Internet-mediated walking program for people with chronic illness. SUH is a multi-component, theory-based physical activity intervention that uses uploading pedometers to objectively monitor and track daily walking and to set personalized goals [21,22]. SUH was specifically designed to help sedentary patients diagnosed with type 2 diabetes, coronary artery disease, or obesity become physically active and reduce their cardiovascular disease risk. The program focuses on home-based unsupervised walking, a low-risk mode of physical activity that can be performed without direct clinical supervision even in high-risk patients [8]. SUH increased walking among sedentary individuals with diabetes by an average of approximately 1 mile or 20 minutes of walking per day over a six-week period [21]. These high-risk individuals are precisely the patient population that is most likely to derive the greatest benefit from moderate increases in activity through reduced cardiovascular mortality and morbidity [23]. However, like many promising Internet-based physical activity programs, SUH has not been translated into a clinical tool that could enable providers to effectively promote physical activity as part of routine care.

Clinical tools for primary care practice that improve efficient resourcing and patient outcomes are fundamental to the patient-centered medical home (PCMH) model of care. The PCMH model aims to improve patient outcomes through the use of technological innovations, shared responsibilities among collaborative provider teams (e.g., physicians, nurses, physician assistants, and medical assistants), and personally-tailored preventive health programs delivered at a population level [24,25]. While Internet-based patient self-management programs offer the benefits of reaching many patients at low cost, they must first be integrated into clinical practice through iterative testing and refinement with provider input [26,27]. The potential of SUH to help PCPs achieve PCMH goals is limited if PCPs do not find SUH easy to use in combination with other key clinical tools such as the electronic medical record (EMR).

This pilot study was designed to accomplish two objectives: 1) to develop a clinical interface with provider input that allows PCPs to efficiently refer patients to SUH and to serially monitor their progress and safety; and 2) to evaluate the feasibility of incorporating the SUH intervention into clinical practice using qualitative interviews, process and satisfaction measures and objective measures of patient physical activity via daily pedometer step counts. The design and implementation of the clinical interface for SUH was driven by the principles of the PCMH model to improve patient-centered care and outcomes through the use of an *e*-health technology accessible by practice care teams. This pilot study was funded through the University of Michigan's Institute for Clinical & Health Research (UL1RR024986), which is funded by a National Institutes of Health Clinical and Translational Sciences Award program.

## Methods

We used a two-phased, mixed-method design to: 1) develop an online clinical interface allowing providers to refer patients to the SUH intervention and to monitor their program compliance; and 2) evaluate the feasibility of implementing SUH in clinical practice. This study was approved by the University of Michigan Institutional Review Board (HUM00016019), and both provider and patient participants completed an informed consent process.

### Phase I

#### Sample

We recruited six pairs of physicians (n = 6) and medical assistants (MAs; n = 7) from two family medicine clinics in a large healthcare system. These provider pairings were representative of local clinical processes of care. All providers gave written informed consent and received a $150 honorarium, a SUH t-shirt, a water bottle, and a one-year membership to WalkingSpree.com, a commercial Internet walking program.

#### Clinical interface development

We employed an *iterative *design process to elicit provider input into the features of the clinical interface [28,29]. Providers were asked to use the SUH intervention for *at least *one week with an emphasis on the features for enrolling patients into the program. Subsequently, investigators DEG and LRB conducted semi-structured, 30-45 minute interviews ("Interview 1") to understand provider attitudes about SUH usability, and recommendations for the interface. During each interview, the interviewer guided the provider participant through existing or demonstration versions of potential web pages to elicit provider needs for key features that would facilitate use. Questions and probes in the semi-structured interview were developed by our research team, and interviews were recorded and transcribed for later analysis of key themes.

Based on provider feedback, the study team worked with computer programmers and an expert in medical informatics to develop automated features necessary for providers to refer patients to the existing SUH participant program and to track their progress. Paper mock-ups of the interface were presented to providers for review, and additional feedback was gathered for interface refinement (in "Interview 2") followed by further interface development. A key component of qualitative analysis is "member checking" in which research team interpretation of interview content is reviewed with participants to clarify and ensure accuracy. Feedback from providers served as a member check for the accuracy of the interpretation of Interview 1 results and as a check that the interface would realistically fit in regular practice work flows (i.e., external validity) [30]. Furthermore, two family physicians on the research team (investigators CRR, CWK) also served as validity checks at each step of the design process to ensure the external validity of features incorporated into the interface.

### Phase II

#### Sample

Each physician-MA pair was asked to recruit 6-8 sedentary adult patients with coronary artery disease, type 2 diabetes, and/or BMI > 25 to participate in the SUH intervention. Patients received a $25 incentive and a one-year WalkingSpree.com membership for completion.

#### Recruitment

**Figure 1:**
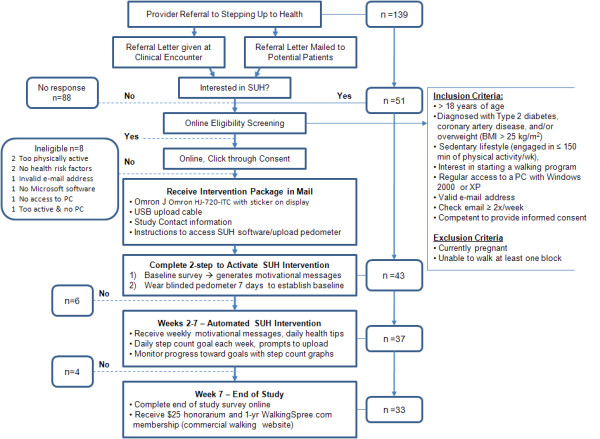
**Intervention and Recruitment Flow**.

As described in Figure 1, providers referred potential patients to the SUH intervention using the newly developed clinical interface. We supplied providers with lists of eligible patients identified from a clinical patient database. Provider referral to the program was considered implicit medical clearance for program participation. Referred patients were directed to an enrollment website that verified their eligibility.

Eligible patients completed an online consent form with a click-through consent process before participating. During the six-week SUH intervention, participants were asked to wear a pedometer, upload step-count data to the SUH server, and review a personalized website with step-count feedback, individualized goals, tailored motivational messages, and educational tips. Patient participants completed an online satisfaction survey during week six of the intervention. Finally, within 2-3 weeks of having all recruited patients complete the intervention, a semi-structured interview was conducted with each provider to assess provider attitudes and perceptions of using the interface with patients as well as beliefs about the broader feasibility of using SUH within their clinical practice ("Interview 3").

### Qualitative Data Analysis

Provider interview data was independently coded by two raters (investigators LRB, DEG) to identify key themes, and a third rater (investigator CRR) helped achieve consensus in cases of disagreement [31]. Identified themes were classified into three categories: 1) comments about the SUH patient intervention; 2) comments regarding the process for recruiting and referring patients; and 3) suggestions for how to design the clinical interface for recruiting patients and tracking their progress in the program. Qualitative results from the first two provider interviews were shared with the research team to inform the clinical interface development. Qualitative results from the third provider interviews were used to evaluate the interface's usability and the feasibility of incorporating the intervention into everyday practice. A fourth member of the research team and provider at one of the research sites (investigator CWK) performed a member check to verify the validity of the conclusions' recommendations for interface development as well as the summary themes that emerged from qualitative analysis.

### Quantitative Data Analysis

Descriptive statistics were used to summarize referral patterns, patient characteristics, and utilization patterns of the clinical interface. Paired t-tests were used to compare patient baseline and end-of-intervention step counts. Details of step-count analysis including methods of handling missing data have been described previously [21,32]. Quantitative data analysis was performed using STATA 10.1 (StataCorp, College Station, Texas).

## Results

### Phase I

#### Recommendations for clinical interface capabilities

Several themes emerged (see Table 1) regarding features that providers desired in a clinical interface. These included notification alerts for individuals who consistently fell short of their program goals over weeks or months, for exercise-related adverse events, or for occasions where the patient consistently failed to upload his or her pedometer over a pre-specified period of time. Additional comments focused on the intervention's capacity for electronic follow-up (i.e., a reminder to view patient data or to send emails to patients). Some providers wanted to view all of the SUH patients within a specific practice and to integrate different types of data logging such as blood pressure, weight, caloric intake, and glucose. Finally, providers indicated a preference for access to printable summary reports for oversight and use during clinical encounters.

**Table 1:** Qualitative data themes elicited from provider interviews about the interface

Qualitative Theme	Exemplary Quote
Phase I	
*The SUH Intervention*	• "I like the site." • "Very slick. It's very impressive." • "I found it pretty easy to use." • "I liked the graphs. I think the visuals were useful." • "I think the graphs are excellent the way they are. It's simple. It explains it. It nails it down to a T."
*Recruitment & Referring*	• "A hand out of some sort is helpful. Just like a single page, here's what it's about, here's the website." • "Some advertisements. Some posters out front. Something that might motivate the patient while they're waiting to be called back to the room."
*Interface Capabilities*	• "...it would be helpful to have something that my medical assistant or somebody in the office could print out on the day that the patient was coming in and say this is what their steps have been doing and so I didn't have to log in necessarily or have them upload any pedometer information at the visit just to make it as quick as possible to go through."

**Phase II**	

*Referral Process*	• "It seemed like the only time that it worked was if it was a face-to-face initiation from we [*sic*] offering it. The letters that we sent out...no one took notice..."
*Impact on Workflow*	• "It would be nice if I'd had [MA] ... kind of do it for me. But if there was any way that I could link up with the patient record I'm looking at because essentially if it pulls the data from [EMR] than this is all filled out for me basically and I just pick the yes, no - this is an acceptable patient for a walking trial and click send, and it is done."
*Patient Monitoring*	• "...just remember that it exists. I did not remember. I had the [study] fliers. I had like the thing put in front of my face on the bulletin board but we're so focused on the computer. I guess if I had opened the website every day then it would trigger me to remember it but I didn't remember it most of the time."
*Interface Usability*	• "It's a great tool. It's not necessarily a time saver. It just gives me a lot more information than I otherwise would get. Real information, not the patient's perception of their own exercise when I see them once a year or four times a year for their follow up. It's real hard data which is very helpful..."
*Barriers to Use*	• "So in that short 6-7 weeks span, the chance of seeing one of these patients in the office while they're doing it is pretty limited and that's really when I'm most prompted to pull up their data and look at it." • "...it would kind of be nice if the doctors included us [MAs] more in it...Kind of give us feedback about some of the adverse reaction emails that they [patients] had or... touch base so that we knew what was going on with the patients ...So that we didn't just refer them, and then they were kind of just out there..."

#### Recommendations for recruitment and referral

Providers specified they would like posters, brochures, and/or other print-based handouts to distribute to patients for recruitment and to provide more program details. Despite the proposed ability of the clinical interface to monitor program participation, many providers expressed concern that motivating individuals to start and stay compliant with SUH would be a challenge: *"The big part of it is just having something to get people motivated and doing it."*

#### Clinical interface innovations

Based on provider input, we developed a clinical interface that included the following pages (see additional file 1 - SUH Clinical Interface Screenshots):

##### Patient Referral Page

Upon entering a patient's name and identification number, providers could automatically generate a personalized recruitment letter containing basic information about the intervention, research study, and enrollment procedures. This letter could be distributed to the patient during a clinical encounter or mailed to the patient.

##### Clinic Summary Page

Providers could view a list of patients enrolled in the study; which included summary information about the status of each patient regarding completion of baseline enrollment criteria and progress in the intervention phase of the program.

##### Detailed Patient Page

Providers could view detailed patient walking data including a graph of daily step counts and weekly step-count goals. Additional features included a list of any illnesses and/or injuries reported by a participant, as well as active hyperlinks for providers to directly email patients or the study team.

We also developed non web-based materials including posters and flyers to aid providers with patient recruitment.

### Phase II

#### Clinical referral process (*see *Table 1* for themes elicited from provider interviews*)

Providers generated 139 referral letters that were mailed or distributed to patients at appointments. Overall, 37 of the referred patients (27%) began the intervention with 34 of the patients (92%) completing the program (Figure 1).

The majority of provider pairs reported that the referral page was simple and straightforward. However, some physicians indicated that it was often difficult to fit a referral into a clinical visit due to competing priorities. In addition, lack of knowledge and comfort with physical activity counseling was cited as a barrier to referral.

#### Impact of referrals on clinic workflow

Physician-MA pairs referred patients during office visits or by mail, with both strategies requiring additional time for coordinating and implementing the referral process. Several pairs found success when physicians discussed physical activity with patients during clinical encounters, offering SUH as a way to increase physical activity while MAs completed the referral process.

Physicians typically viewed referrals as an additional time burden, with one physician commenting: *"I'm trying to do so many things ...I never figured out how to fit it in." *In contrast, MAs viewed the referral process as more feasible: *"...We always have another thing added on. It's just like any other thing...So I mean an extra couple minutes...you really don't notice it after a while after you get used to doing it."*

#### Implementation of the clinical interface for patient monitoring

The frequency of use of the clinical interface for monitoring patient progress was low. Website usage logs showed that the Clinic Summary Page was viewed a total of 100 times across all provider participants. The Detailed Patient Pages were viewed for only 15 of the 37 participants for a total of 23 page views.

Providers mentioned that because SUH was not integrated into their electronic medical record (EMR) system, accessing the program was not convenient as it required taking time during a clinical visit to find the website and remember their username and password. Many providers noted that they lacked a visual cue to login to the interface.

Once providers logged into the interface, feedback regarding usability was positive. Providers found the Detailed Patient Page easy to use and reported *"enough basic info" *was presented to quickly check a patient's progress. Both physicians and MAs felt that this was functional, informative, and that graphical display of patient walking progress was effective.

Several barriers to interface use were attributed to the short duration of the pilot intervention, which made it more difficult to integrate it into normal processes of care. Some providers requested email prompts to review specific patient records for situations that could necessitate provider follow-up or encouragement. These situations could include the occurrence of a medical event affecting walking, low program participation, or a scheduled clinical encounter. Moreover, some MAs observed that once they assisted with the referral phase of the program, they were largely left out of monitoring patient progress, even though they were more likely to interact with patients between routine visits. Finally, lack of time to cover physical activity discussions during brief patient visits was a ubiquitous barrier to effectively using the interface.

#### Patient results

The 37 patients who began the SUH intervention had a mean age of 45.2 (SD = 9.9) and were predominantly female, white, clinically obese, and well-educated (Table 2). Almost two-thirds of patients had never used a pedometer, and 54% described their Internet proficiency as basic or moderate.

**Table 2:** Baseline socio-economic and health characteristics for patient participants

N	37
Age (SD)	45.2 (9.9)
Race, %^a^	
American Indian	5.4
Black	5.4
White	91.9
Female, %	65.0
Education,%	
High School degree, GED, or less	8.2
Some College or 2-year degree	45.9
College or graduate degree	45.9
Income, %	
< $30,000	13.5
$30,000 - $49,999	24.3
$50,000 - $69,999	16.2
> $70,000	46.0
Marital status, %	
Married	31
Divorced	2
Single	4
Other	2
Internet Experience, %	
Basic	16.2
Moderate	37.8
Advanced	35.2
Expert	10.8
Previous Pedometer Use,%	
Yes	37.8
No	62.2
BMI (kg/m^2^)	40.7 (7.6)
Currently Smoking, %	3 (8.1%)
Diagnosis,%	
Obese^b^	97.3%
Type 2 Diabetes	21.6%
Coronary Artery Disease	8.1%

^a ^Some participants self-identified as more than one race

^b ^100% of sample was overweight (BMI > 25) with n = 1 reporting BMI < 30

Intent-to-treat analyses of the 37 participants enrolled in the SUH intervention showed the program was effective in helping this high-risk sample become more physically active. Paired *t*-tests showed that participants significantly increased their total daily average steps from baseline (*M *= 4520, *SD *= 309) to post-intervention (*M *= 6013, *SD *= 443), for an average increase of 1493 steps (*t*(36) = 4.13, *p *< 0.01) (Table 3). Total minutes of aerobic walking per week also increased significantly from baseline (*M *= 27.0. *SD *= 8.8) to post-intervention (*M *= 71.7, *SD *= 14.7) by an average of 44.7 minutes per week (*t*(36) = 2.96, *p *< 0.01). Most of the users found the intervention useful and easy to use (Table 3).

**Table 3:** Indicators of program effectiveness

Changes in Patient Step Counts (N = 37)	Baseline Week	Final Week	*p *value
Average total daily steps	4520 ± 309	6013 ± 443	*p *= 0.0002
Average daily aerobic steps	406 ± 137	1114 ± 218	*p *= 0.0026
Minutes of aerobic walking/week ^a^	27.0 ± 8.8	71.7 ± 14.7	*p *= 0.0054

**Patient Satisfaction^b ^(N = 34)**	**Mean (SD)**	**Agree or strongly agree**	

SUH is useful	4.6 (0.7)	94.1%	
SUH increases the amount I walk	4.6 (0.9)	85.3%	
SUH is easy to use	4.7 (0.6)	94.1%	
I like working with SUH	4.6 (0.7)	88.2%	

^a ^Aerobic minutes of ambulatory activity are recorded by the Omron HJ-720-ITC pedometer. According to the criteria, data are recorded when an individual continuously engages in continuous movement such as walking, jogging, or running at a rate of 60^+ ^steps per minute, involves no more than a 60-second pause, and lasts at least 10 minutes in duration.

^b ^Satisfaction scores are based on a 5-item Likert scale where 5 = *strongly agree *and 1 = *strongly disagree*.

## Discussion

Results demonstrate the feasibility of using an online clinical tool to enable PCPs to promote physical activity in high-risk patients. The developed clinical interface is a promising approach to help reorient primary care providers on lifestyle factors such as physical activity promotion [33-35]. The PCMH concept of care combines information technology tools with a collaborative team approach to provide more time for services like physical activity counseling. Programs like SUH help support the PCMH concept of care by emphasizing the efficient application of information technology to facilitate coordination between patients and their providers [24]. SUH supports patients with personalized walking goals and feedback, facilitates ongoing patient-provider communication, and gives PCPs the ability to prospectively monitor patient progress and safety.

The SUH intervention and clinical interface have several advantages over previous strategies used to promote lifestyle change in primary care. One advantage is that the automated referral process requires minimal provider time and can reach large numbers of patients with a consistent approach [36] in contrast to more traditional strategies such as face-to-face provider counseling [37-39] or referral by clinical staff to community programs [33,40,41]. A second advantage is that the clinical interface gives clinical providers ongoing feedback and monitoring regarding the progress of patients' walking programs. This second strength is further enhanced by the capability of patients and providers to email each other as needed so that the intervention is not disconnected from clinical practice after referral [41,42]. This is consistent with the PCMH model of care in that the primary health care team remains informed about all aspects of care.

The clinical interface provided PCPs with the option of either directly referring patients to SUH during a clinical encounter or using a clinical database to identify eligible patients to send a referral letter by mail. Over 25% of the patients referred to SUH were enrolled despite some providers expressing lack of confidence in their physical activity counseling skill. This lack of confidence is a known barrier to successful implementation of practice-based physical activity interventions [43,44]. It is possible that training programs for providers that directly address physical activity counseling skills may increase program adoption by patients. Nonetheless, use of a physical activity program by 25% of a practice network's high-risk patients could yield a significant population effect on morbidity and mortality, service utilization, and patient quality of life and optimal function.

Although providers were successful in referring patients to the program, they rarely used the clinical interface to monitor patients enrolled in SUH. This may have been due to the short duration of the follow-up period, lack of time for providers, and lack of integration of the clinical interface into the clinics' EMR infrastructure. Prior studies of automated clinical tools to support behavior change have noted that full integration of these tools with the practice EMR is desirable but challenging. Integration allows efficient access information for patient monitoring and consultation [45].

Implementation of SUH could be improved by emphasizing a team-based approach to patient referral and by ongoing program monitoring. Involving MAs more regularly in participant monitoring might have strengthened the effect on patient adoption and use of SUH. Time constraints faced by physician providers necessitate greater reliance on nurses and MAs [24]. Furthermore, evidence suggests health behavioral change interventions are more effective when allied health personnel are trained to augment physician brief counseling and education efforts [46-49].

While the focus of this study was on testing the feasibility of the SUH as a clinical practice tool [50], Phase II testing revealed encouraging preliminary patient outcome results. Referred patients who chose to participate in the walking program significantly increased their physical activity over six weeks from an average of 45 min/wk to 72 min/wk. Despite the automated nature of the SUH walking program, participants reported high levels of satisfaction with the program. As demonstrated in previous walking studies, high-risk patients were able to successfully participate without experiencing serious adverse events [51].

This study used a combination of evaluation methods suited to assessing the feasibility of implementing an intervention in real world settings [52]. First, this study employed an iterative, user-centered design process to ensure that the clinical interface met the needs of the providers [28]. Multiple qualitative interviews with providers identified user needs and barriers to implementation that could not be easily understood using quantitative data alone [29,53]. Second, we recognized the competing priorities and time limitations faced by providers during clinical encounters and automated the SUH referral processes to minimize PCP burden. Finally, to focus on implementation rather than efficacy, there was minimal contact between researchers and provider teams or patients during Phase II testing. Thus, provider pairs tested the system in the context of their regular clinical workflow.

### Limitations

There are several study limitations worth noting. First, the study did not utilize a randomised controlled trial design with comparison to standard-of-care control participants. As previously noted, the developmental costs of many *e*-health technological interventions necessitate the use of smaller pilot studies as part of iterative research in preparation of a randomised, controlled comparative effectiveness trial (RCT). An RCT establishes clinical efficacy and cost-effectiveness in defined patient groups, but this approach was beyond the scope and aims of the current study. Notably, the current study enabled the development of a robust provider interface that lays the groundwork for SUH to be comparatively evaluated in a future RCT. Second, the short duration of this pilot intervention did not reflect real world practice where patients would be monitored over months or years versus 6-7 weeks. The short duration of the intervention may have limited provider motivation to monitor participant progress. However, the primary emphasis of this pilot study was to develop a robust clinical interface and to establish feasibility with a small sample of end users (e.g., clinical staff and patients). Third, it is also worth noting that the study did not assess changes in physiological outcomes and did not assess practical screening measures to facilitate referral to SUH. Physiological outcomes such as changes in blood pressure, cholesterol or blood glucose are key indicators of self-management outcomes. However, outcomes in this study focused on indicators of satisfaction and program engagement by the end users.

In the present study, provider pairs were engaged in the interface design process during Phase I and reviewed proposed interface implementation strategies. The providers had fewer opportunities to provide input into workflow integration issues once the interface was developed. This is a significant and fourth limitation as workflow concerns and, in particular, concerns about increased workload were frequently mentioned by providers. PCMH efforts to reorganize processes of care and improve productivity through technological innovations may have unintended consequences by actually creating additional work or causing provider dissatisfaction particularly if providers do not have the opportunity to influence practice redesign efforts [54].

Results from this study provide a foundation to build on in future investigations. In particular, more work is needed to ensure that the interface is flexible enough to accommodate a broad range of variation in workflow patterns and to ensure intervention protocols are acceptable and feasible to providers.

As a fifth limitation, providers also complained that SUH was not part of the clinical EMR. Ideally, research intervention programs like SUH could be tested as part of the EMR and embedded with all patient medical information. This association could allow rapid comparison of program progress with changes in physiological outcomes (e.g. hemoglobin A1_c_, lipids). In addition, electronic patient visit prompts could remind providers to inquire about program walking progress. Finally, this study sample was a small group of volunteer patients from only two family medicine clinics, which limits generalization and the statistical power of our analyses to examine the influence of factors such as patient characteristics (e.g. prior pedometer use) on the results.

## Conclusions

Primary care providers can successfully refer patients to an Internet-mediated walking program using a web-based interface. Patients who chose to enroll in the program significantly increased their walking. However, providers rarely used the interface to monitor patient progress in the program. A number of barriers to provider use of the interface were identified during the program. The results of this study will enable the research team to conduct a large randomised-clinical trial to compare program results with other intervention strategies (e.g., phone, face-to-face counseling) and to evaluate the prospects for large-scale implementation of the SUH program in clinical practice.

## List of abbreviations

SUH: Stepping Up to Health; PCPs: primary care physicians; PCMH: patient-centered medical home; NIH: National Institutes of Health; EMR: electronic medical record; MAs: medical assistants; RCT: Randomised controlled trial.

## Competing interests

The authors declare that they have no competing interests. Dr. Richardson is a scientific advisor for WalkingSpree.com but does not receive any compensation.

## Authors' contributions

DEG and CRR designed and developed the study, obtained funding, oversaw the implementation of the intervention, analyzed the data, and wrote the manuscript.

LRB assisted in the development of surveys and interview guides, acquisition of data, analysis and interpretation of qualitative data, and wrote the manuscript.

AWJ was the web interface development manager, assisted with data analysis, and reviewed the manuscript.

MDD assisted in the acquisition of data, analysis and interpretation of qualitative and quantitative data, and assisting in writing the manuscript.

CWK helped with research site recruitment, provided a member check to the interpretation of qualitative findings, and reviewed many drafts of the final version of the manuscript.

VJS obtained funding, provided technical support in the development of the computerized intervention platform, and reviewed multiple drafts of the final version of the manuscript.

KZ provided expert consultation on the development of provider needs assessment interview guides, interpretation of qualitative data for development of the clinical software interface, and reviewed multiple drafts of the final manuscript.

AS obtained funding, assisted in the design, analysis, and interpretation of quantitative data, and reviewed the final version of the manuscript.

MLH oversaw the development of the clinical interface, oversaw online data acquisition, and reviewed the final version of the manuscript.

JDP obtained funding and reviewed multiple versions of the manuscript.

All authors read and approved the final manuscript.

## Pre-publication history

The pre-publication history for this paper can be accessed here:

http://www.biomedcentral.com/1472-6947/11/47/prepub

## Supplementary Material

Additional file 1 **SUH Clinical Interface Screenshots**. Screenshots of Stepping Up to Health Clinical Interface. Click here for file

## References

[B1] American Diabetes Association Standards of medical care in diabetes-2010 Diabetes Care 2010 33 Suppl 1 S11 61 20042772 10.2337/dc10-S011 PMC2797382

[B2] De Hert M Dekker JM Wood D Kahl KG Holt RI Moller HJ Cardiovascular disease and diabetes in people with severe mental illness position statement from the European Psychiatric Association (EPA), supported by the European Association for the Study of Diabetes (EASD) and the European Society of Cardiology (ESC) European Psychiatry 2009 24 412 424 10.1016/j.eurpsy.2009.01.005 19682863

[B3] Doyle C Kushi LH Byers T Courneya KS Demark-Wahnefried W Grant B Nutrition and physical activity during and after cancer treatment: an American Cancer Society guide for informed choices CA Cancer J Clin 2006 56 323 353 10.3322/canjclin.56.6.323 17135691

[B4] Eyre H Kahn R Robertson RM Clark NG Doyle C Hong Y Preventing cancer, cardiovascular disease, and diabetes: a common agenda for the American Cancer Society, the American Diabetes Association, and the American Heart Association Circulation 2004 109 3244 3255 10.1161/01.CIR.0000133321.00456.00 15198946

[B5] Pi-Sunyer X Blackburn G Brancati FL Bray GA Bright R Clark JM Reduction in weight and cardiovascular disease risk factors in individuals with type 2 diabetes: one-year results of the Look AHEAD trial Diabetes Care 2007 30 1374 1383 17363746 10.2337/dc07-0048 PMC2665929

[B6] Slentz CA Duscha BD Johnson JL Ketchum K Aiken LB Samsa GP Effects of the amount of exercise on body weight, body composition, and measures of central obesity: STRRIDE--a randomized controlled study Arch Intern Med 2004 164 31 39 10.1001/archinte.164.1.31 14718319

[B7] Knowler WC Barrett-Connor E Fowler SE Hamman RF Lachin JM Walker EA Reduction in the incidence of type 2 diabetes with lifestyle intervention or metformin N Engl J Med 2002 346 393 403 11832527 10.1056/NEJMoa012512 PMC1370926

[B8] Franklin BA Swain DP Shephard RJ New insights in the prescription of exercise for coronary patients J Cardiovasc Nurs 2003 18 116 123 12680570 10.1097/00005082-200304000-00007

[B9] Fletcher GF Balady GJ Amsterdam EA Chaitman B Eckel R Fleg J Exercise standards for testing and training: a statement for healthcare professionals from the American Heart Association Circulation 2001 104 1694 1740 10.1161/hc3901.095960 11581152

[B10] Murphy MH Nevill AM Murtagh EM Holder RL The effect of walking on fitness, fatness and resting blood pressure: a meta-analysis of randomised, controlled trials Prev Med 2007 44 377 385 10.1016/j.ypmed.2006.12.008 17275896

[B11] Estabrooks PA Glasgow RE Translating effective clinic-based physical activity interventions into practice Am J Prev Med 2006 31 Suppl 4 S45 56 16979469 10.1016/j.amepre.2006.06.019

[B12] Eden KB Orleans CT Mulrow CD Pender NJ Teutsch SM Does counseling by clinicians improve physical activity? A summary of the evidence for the U.S. Preventive Services Task Force Ann Intern Med 2002 137 208 215 12160371 10.7326/0003-4819-137-3-200208060-00015

[B13] Jacobson DM Strohecker L Compton MT Katz DL Physical activity counseling in the adult primary care setting: position statement of the American College of Preventive Medicine Am J Prev Med 2005 29 158 162 16005814 10.1016/j.amepre.2005.04.009

[B14] Cress ME Buchner DM Prohaska T Rimmer J Brown M Macera C Best practices for physical activity programs and behavior counseling in older adult populations J Aging Phys Act 2005 13 61 74 15677836 10.1123/japa.13.1.61

[B15] Douglas F Torrance N van Teijlingen E Meloni S Kerr A Primary care staff's views and experiences related to routinely advising patients about physical activity. A questionnaire survey BMC Public Health 2006 6 138 10.1186/1471-2458-6-138 16719900 PMC1523207

[B16] Castaldo J Nester J Wasser T Masiado T Rossi M Young M Physician attitudes regarding cardiovascular risk reduction: the gaps between clinical importance, knowledge, and effectiveness Dis Manag 2005 8 93 105 10.1089/dis.2005.8.93 15815158

[B17] Wee CC McCarthy EP Davis RB Phillips RS Physician counseling about exercise JAMA 1999 282 1583 1588 10.1001/jama.282.16.1583 10546701

[B18] Napolitano MA Marcus BH Targeting and tailoring physical activity information using print and information technologies Exerc Sport Sci Rev 2002 30 122 128 10.1097/00003677-200207000-00006 12150571

[B19] Kuhl EA Sears SF Conti JB Internet-based behavioral change and psychosocial care for patients with cardiovascular disease: a review of cardiac disease-specific applications Heart Lung 2006 35 374 382 10.1016/j.hrtlng.2006.02.004 17137938

[B20] Winett RA Tate DF Anderson ES Wojcik JR Winett SG Long-term weight gain prevention: a theoretically based Internet approach Prev Med 2005 41 629 641 10.1016/j.ypmed.2004.12.005 15917062

[B21] Richardson CR Mehari KS McIntyre LG Janney AW Fortlage LA Sen A A randomized trial comparing structured and lifestyle goals in an internet-mediated walking program for people with type 2 diabetes Int J Behav Nutr Phys Act 2007 4 59 10.1186/1479-5868-4-59 18021411 PMC2212636

[B22] Richardson CR Buis LR Janney AW Goodrich DE Sen A Hess ML An online community improves adherence in an internet-mediated walking program. Part 1: results of a randomized controlled trial J Med Internet Res 2010 12 e71 21169160 10.2196/jmir.1338 PMC3056526

[B23] Richardson CR Kriska AM Lantz PM Hayward RA Physical activity and mortality across cardiovascular disease risk groups Med Sci Sports Exerc 2004 36 1923 1929 10.1249/01.MSS.0000145443.02568.7A 15514508

[B24] Crabtree BF Nutting PA Miller WL Stange KC Stewart EE Jaen CR Summary of the National Demonstration Project and recommendations for the patient-centered medical home Ann Fam Med 8 Suppl 1 S80 90 10.1370/afm.1107 PMC2885727 20530397

[B25] Jaen CR Ferrer RL Miller WL Palmer RF Wood R Davila M Patient outcomes at 26 months in the patient-centered medical home National Demonstration Project Ann Fam Med 8 Suppl 1 S57 67 10.1370/afm.1121 PMC2885729 20530395

[B26] Griffiths F Lindenmeyer A Powell J Lowe P Thorogood M Why are health care interventions delivered over the internet? A systematic review of the published literature J Med Internet Res 2006 8 e10 10.2196/jmir.8.2.e10 16867965 PMC1550698

[B27] Bennett GG Glasgow RE The delivery of public health interventions via the Internet: actualizing their potential Annu Rev Public Health 2009 30 273 292 10.1146/annurev.publhealth.031308.100235 19296777

[B28] Beyer H Holtzblatt K Contextual Design: Defining Customer-Centered Systems 1998 San Francisco: Morgan Kaufmannn Publishers

[B29] Dumas JS Redish JC A Practical guide to Usability Testing, Revised Edition 1999 Portland: Intellect Books

[B30] Schwartz-Shea P Yanow D, Schwartz-Shea P Evaluative criteria and epistemic communities Interpretation and method: empirical research methods and interpretive turn 2006 Armonk: M.E. Sharpe, Inc 89 113

[B31] Forman J Damschroder LJ Jacoby L, Siminoff L Qualitative content analysis Empirical research for bioethics: A primer 2008 Oxford: Elsevier Publishing 221

[B32] Buis LR Janney AW Hess ML Culver SA Richardson CR Barriers encountered during enrollment in an internet-mediated randomized controlled trial Trials 2009 10 76 10.1186/1745-6215-10-76 19698154 PMC2744913

[B33] Cohen DJ Tallia AF Crabtree BF Young DM Implementing health behavior change in primary care: lessons from prescription for health Ann Fam Med 2005 3 Suppl 2 S12 19 16049075 10.1370/afm.334 PMC1466976

[B34] Hung DY Improving the delivery of preventive care services Manag Care Interface 2007 20 38 44 17626591

[B35] Lloyd-Jones DM Hong Y Labarthe D Mozaffarian D Appel LJ Van Horn L Defining and setting national goals for cardiovascular health promotion and disease reduction: the American Heart Association's strategic Impact Goal through 2020 and beyond Circulation 2010 121 586 613 10.1161/CIRCULATIONAHA.109.192703 20089546

[B36] Marcus BH Ciccolo JT Sciamanna CN Using electronic/computer interventions to promote physical activity Br J Sports Med 2009 43 102 105 19052143 10.1136/bjsm.2008.053744 PMC5718350

[B37] Sciamanna CN Novak SP Marcus BH Effects of using a computer in a doctor's office on patient attitudes toward using computerized prompts in routine care Int J Med Inform 2005 74 357 365 10.1016/j.ijmedinf.2005.03.003 15893258

[B38] Sinclair J Lawson B Burge F Which patients receive advice on diet and exercise? Do certain characteristics affect whether they receive such advice? Can Fam Physician 2008 54 404 412 18337535 PMC2278358

[B39] Goldstein MG Pinto BM Marcus BH Lynn H Jette AM Rakowski W Physician-based physical activity counseling for middle-aged and older adults: a randomized trial Ann Behav Med 1999 21 40 47 10.1007/BF02895032 18425653

[B40] Holtrop JS Dosh SA Torres T Thum YM The community health educator referral liaison (CHERL): a primary care practice role for promoting healthy behaviors Am J Prev Med 2008 35 Suppl 5 S365 372 18929983 10.1016/j.amepre.2008.08.012

[B41] Williams NH Hendry M France B Lewis R Wilkinson C Effectiveness of exercise-referral schemes to promote physical activity in adults: systematic review Br J Gen Pract 2007 57 979 986 10.3399/096016407782604866 18252074 PMC2084138

[B42] Weidinger KA Lovegreen SL Elliott MB Hagood L Haire-Joshu D McGill JB How to make exercise counseling more effective: lessons from rural America J Fam Pract 2008 57 394 402 18544323

[B43] Aittasalo M Miilunpalo S Stahl T Kukkonen-Harjula K From innovation to practice: initiation, implementation and evaluation of a physician-based physical activity promotion programme in Finland Health Promot Int 2007 22 19 27 10.1093/heapro/dal040 17135327

[B44] Jansink R Braspenning J van der Weijden T Elwyn G Grol R Primary care nurses struggle with lifestyle counseling in diabetes care: a qualitative analysis BMC Fam Pract 11 41 10.1186/1471-2296-11-41 PMC2889883 20500841

[B45] Aspy CB Mold JW Thompson DM Blondell RD Landers PS Reilly KE Integrating screening and interventions for unhealthy behaviors into primary care practices Am J Prev Med 2008 35 Suppl 5 S373 380 18929984 10.1016/j.amepre.2008.08.015

[B46] Tulloch H Fortier M Hogg W Physical activity counseling in primary care: Who has and who should be counseling? Patient Educ Couns 2006 64 6 20 10.1016/j.pec.2005.10.010 16472959

[B47] Ferrer RL Mody-Bailey P Jaen CR Gott S Araujo S A medical assistant-based program to promote healthy behaviors in primary care Ann Fam Med 2009 7 504 512 10.1370/afm.1059 19901309 PMC2775613

[B48] Coleman K Mattke S Perrault PJ Wagner EH Untangling practice redesign from disease management: how do we best care for the chronically ill? Annu Rev Public Health 2009 30 385 408 10.1146/annurev.publhealth.031308.100249 18925872

[B49] Puczynski S Phelps K Wilke A Nagel R Hickey D Badenhop D Collaborative goal setting to improve lifestyle behaviors: lessons learned from NOPCRN Ann Fam Med 2005 3 Suppl 2 S60 62 16049093 10.1370/afm.364 PMC1466967

[B50] Glasgow RE Lichtenstein E Marcus AC Why don't we see more translation of health promotion research to practice? Rethinking the efficacy-to-effectiveness transition Am J Public Health 2003 93 1261 1267 10.2105/AJPH.93.8.1261 12893608 PMC1447950

[B51] Goodrich DE Larkin AR Lowery JC Holleman RG Richardson CR Adverse events among high-risk participants in a home-based walking study: a descriptive study Int J Behav Nutr Phys Act 2007 4 20 10.1186/1479-5868-4-20 17521443 PMC1891313

[B52] Saunders RP Evans MH Joshi P Developing a process-evaluation plan for assessing health promotion program implementation: a how-to guide Health Promot Pract 2005 6 134 147 10.1177/1524839904273387 15855283

[B53] Steckler A McLeroy KR Goodman RM Bird ST McCormick L Toward integrating qualitative and quantitative methods: an introduction Health Educ Q 1992 19 1 8 1568869 10.1177/109019819201900101

[B54] Tufano JT Ralston JD Martin DP Providers' experience with an organizational redesign initiative to promote patient-centered access: a qualitative study J Gen Intern Med 2008 23 1778 1783 10.1007/s11606-008-0761-3 18769981 PMC2585688

