# Supplementary material for: Integrating an internet-mediated walking program into family medicine clinical practice: a pilot feasibility study
Source: BMC Med Inform Decis Mak. 2011 Jun 24;11:47. doi: 10.1186/1472-6947-11-47 (PMC3135495; doi:10.1186/1472-6947-11-47)
Supplement: Additional file 1 — SUH Clinical Interface Screenshots. Screenshots of Stepping Up to Health Clinical Interface. [file 1472-6947-11-47-S1.PDF]

SUH Clinical Interface Screenshots

	Page
Title page	1
Table of contents	1
Provider Login	2
Homepage of SUH Clinical Interface	3
Patient Referral Template	4
Welcome to SUH Screening Page	5
Initial Page to Personalize Assessment of Eligibility	6
Clinic Summary Page	7
Detailed Patient Page	8

Figure 2. Provider Login Page

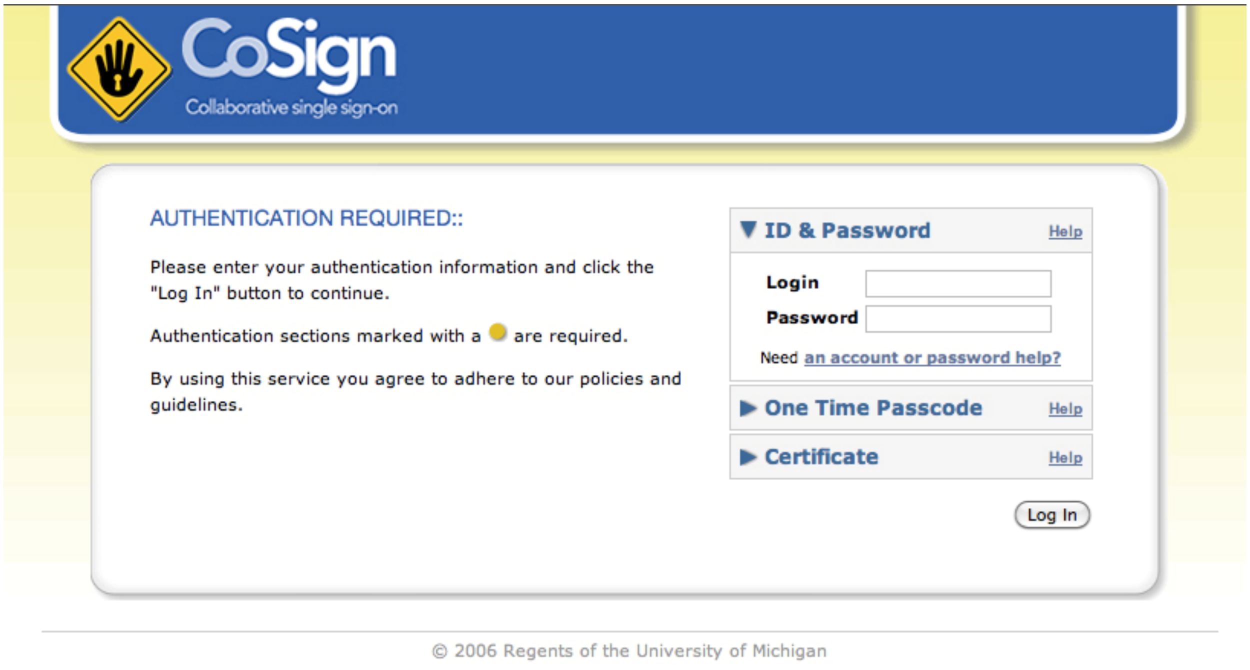

Figure 3. Homepage of SUH Clinical Interface

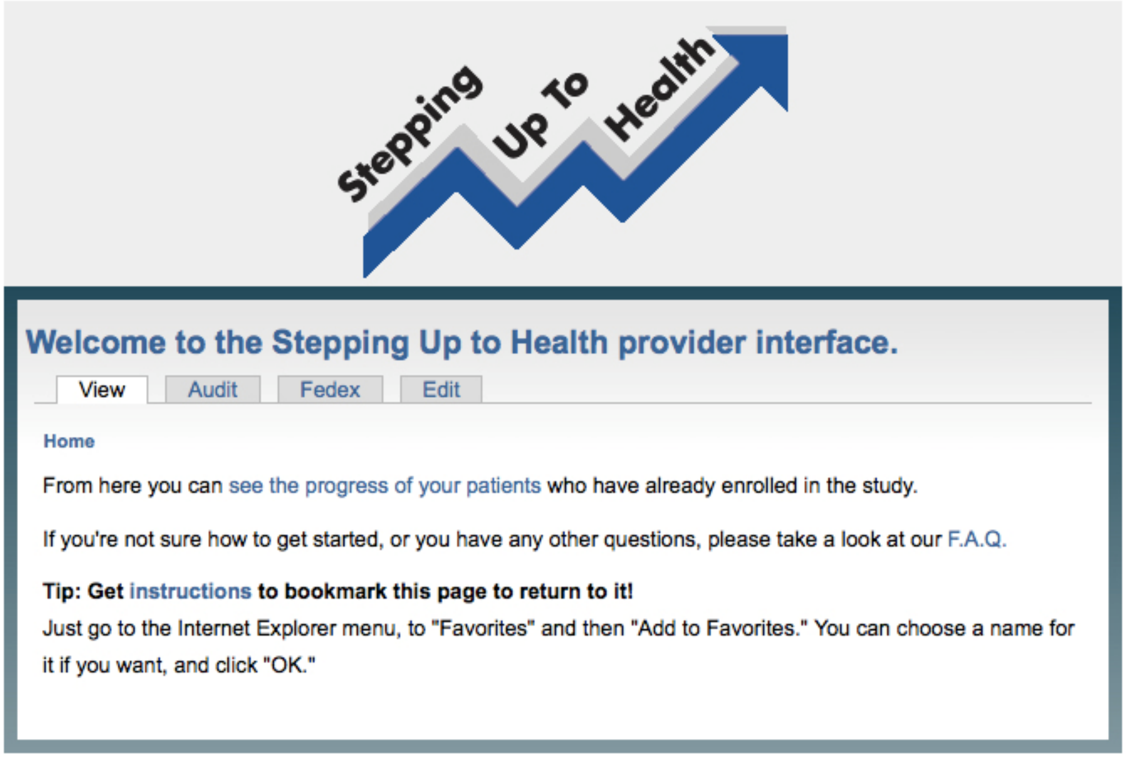

Figure 4. Patient Referral Template

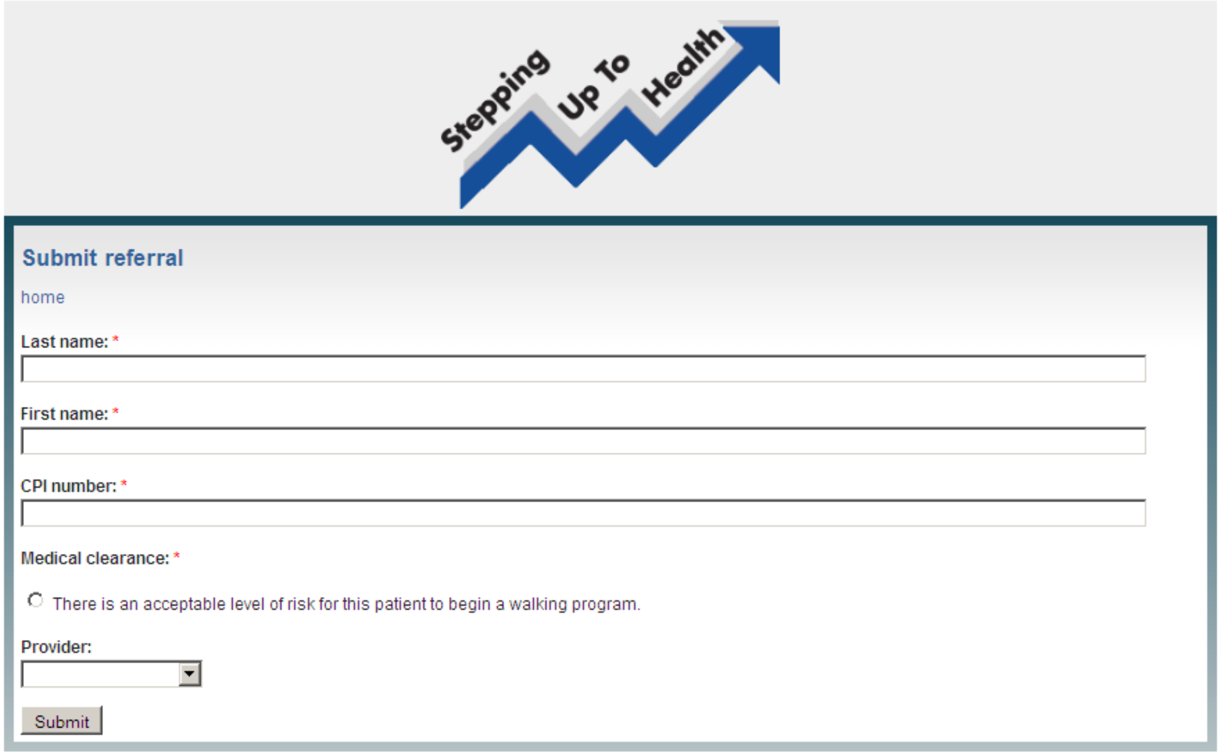

Stepping Up To Health

Submit referral

home

Last name: *

First name: *

CPI number: *

Medical clearance: *

( ) There is an acceptable level of risk for this patient to begin a walking program.

Provider:

Submit

Figure 5. Welcome to SUH Screening Page

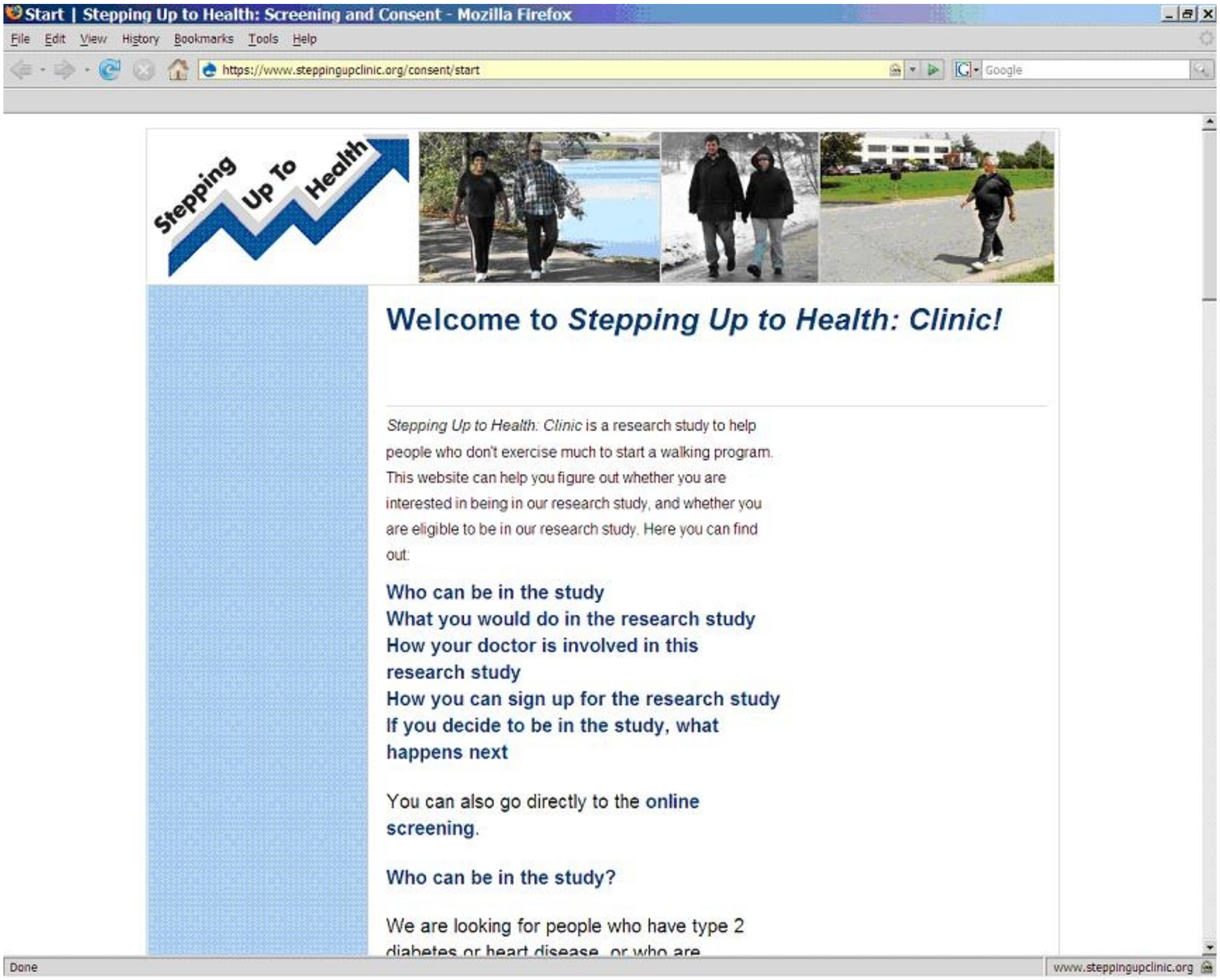

Figure 6. Initial Page to Personalize Assessment of Eligibility Questions

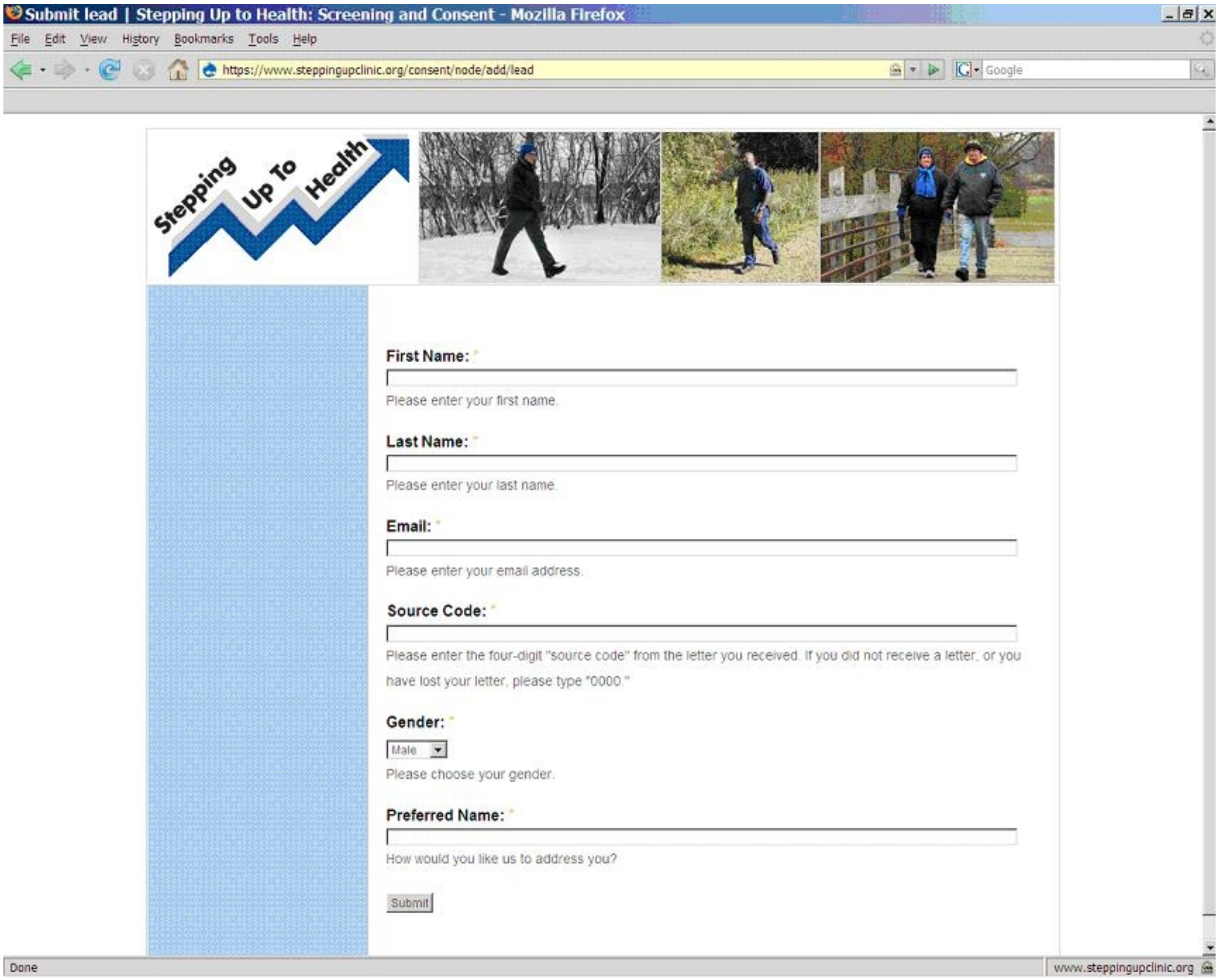

Figure 7. Clinic Summary Page

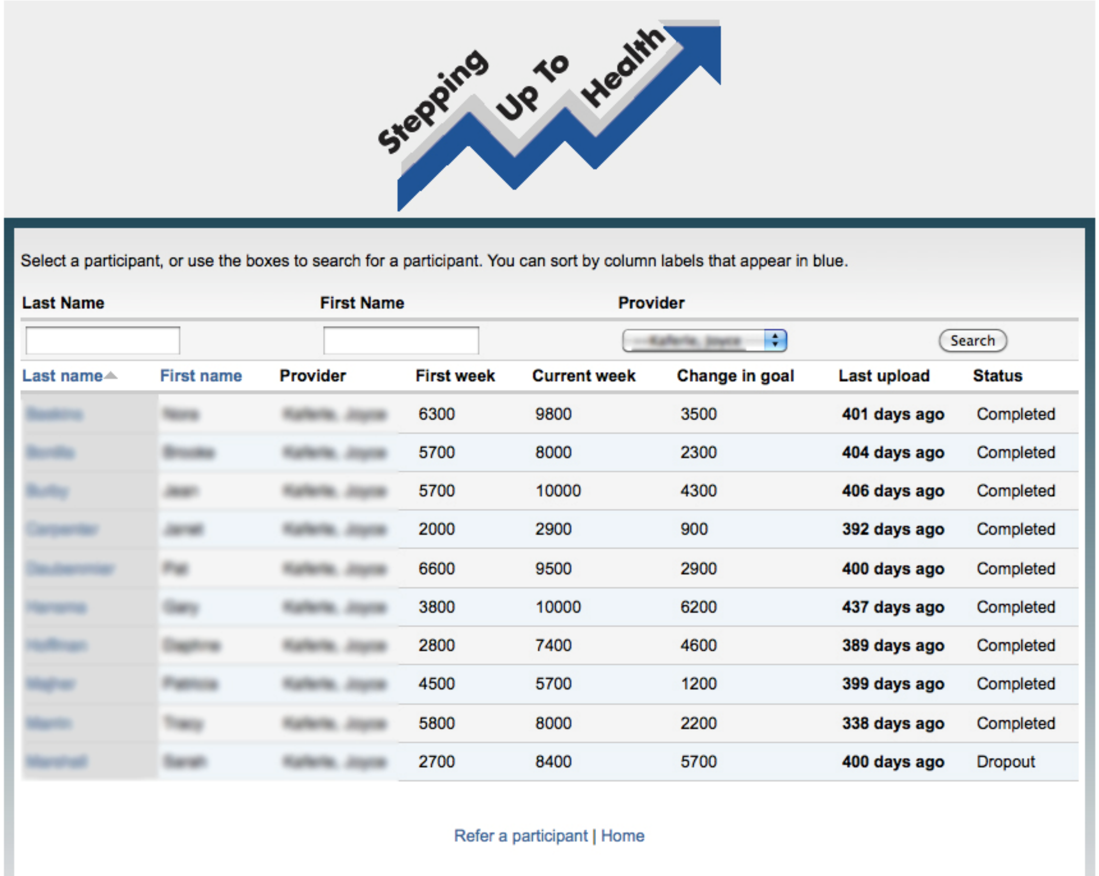

Stepping Up To Health

Select a participant, or use the boxes to search for a participant. You can sort by column labels that appear in blue.

Last Name | First Name | Provider | Search

Last name	First name	Provider	First week	Current week	Change in goal	Last upload	Status
[illegible]	[illegible]	[illegible]	6300	9800	3500	**401 days ago**	Completed
[illegible]	[illegible]	[illegible]	5700	8000	2300	**404 days ago**	Completed
[illegible]	[illegible]	[illegible]	5700	10000	4300	**406 days ago**	Completed
[illegible]	[illegible]	[illegible]	2000	2900	900	**392 days ago**	Completed
[illegible]	[illegible]	[illegible]	6600	9500	2900	**400 days ago**	Completed
[illegible]	[illegible]	[illegible]	3800	10000	6200	**437 days ago**	Completed
[illegible]	[illegible]	[illegible]	2800	7400	4600	**389 days ago**	Completed
[illegible]	[illegible]	[illegible]	4500	5700	1200	**399 days ago**	Completed
[illegible]	[illegible]	[illegible]	5800	8000	2200	**338 days ago**	Completed
[illegible]	[illegible]	[illegible]	2700	8400	5700	**400 days ago**	Dropout

Refer a participant | Home

Figure 8. Detailed Patient Page

Stepping Up To Health

John Doe

Email PT | Email study team | Back to list | Home

Last Upload: 2009-03-06 • Total Uploads: 12

Weekly goals and steps

Week	Avg. steps	Bout steps	Goal	Days goal met
2008-06-15	3999	753	7600	0
2008-06-22	5300	0	7600	1
2008-06-29	1688	634	6800	1
2008-07-06	0	0	6800	0
2008-07-13	0	0	6800	0
2008-07-20	2788	0	6800	1
2008-07-27	3927	1044	6800	2
2008-08-03	1071	0	4000	1
2009-01-18	3249	0	4000	1
2009-01-25	0	0	2700	0
2009-02-01	0	0	2700	0
2009-02-08	5500	1290	2700	4
2009-02-15	3500	0	4200	2
2009-02-22	3356	1503	4200	3
2009-03-01	5785	1656	4200	6

Progress

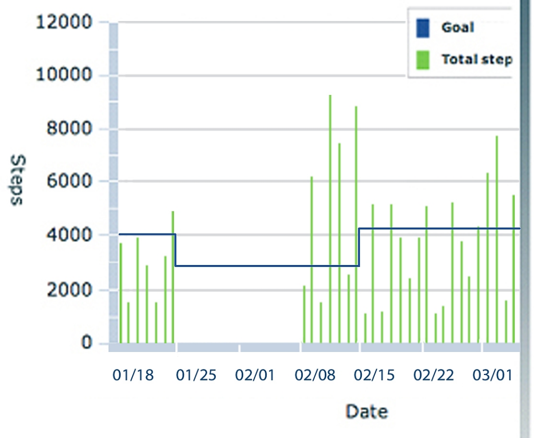

Injuries or illnesses reported to study team

Reported illnesses or injuries take up to 72 hours to appear in this summary list.

Date	Event
